# Coordinated responses to individual tumor antigens by IgG antibody and CD8+ T cells following cancer vaccination

**DOI:** 10.1186/s40425-018-0331-0

**Published:** 2018-04-05

**Authors:** Tyler W. Hulett, Shawn M. Jensen, Phillip A. Wilmarth, Ashok P. Reddy, Carmen Ballesteros-Merino, Michael E. Afentoulis, Christopher Dubay, Larry L. David, Bernard A. Fox

**Affiliations:** 1 Earle A. Chiles Research Institute, Robert W. Franz Cancer Center, Providence Cancer Institute, 2N56 North Pavilion, 4805 NE Glisan St., Portland, OR 97213 USA; 2 Proteomics Shared Resource, Oregon Health & Science University Portland, Oregon, 97239 USA; 3 Department of Biochemistry and Molecular Biology, Oregon Health & Science University Portland, Oregon, 97239 USA; 4 Department of Molecular Microbiology & Immunology, Oregon Health & Science University Portland, Oregon, 97239 USA

**Keywords:** Immunological Monitoring, Antigen, CD8 +, T cell, IgG, Antibody, Vaccine, Autophagosome, Poly-I:C, 4T1

## Abstract

**Background:**

One of today’s greatest hurdles for cancer immunotherapy is the absence of information regarding which tumor antigens are already recognized by patients receiving immunotherapies, and whether those therapies then boost or generate an immune response against tumor proteins. For CD8+ T cells in particular, patient-specific immune recognition and responses at the level of individual tumor antigens are rarely characterized. Because of this, some immunologists have turned to serum antibodies as an alternative measure of antigen-specific anti-tumor immunity. In this work, we sought to simultaneously interrogate serum IgG and CD8+ T cell recognition of individual tumor antigens to determine whether antigen-specific serum IgG antibodies provide a window into the behavior of antigen-specific CD8+ T cell responses. Using antibody-based assays to evaluate immune response repertoires and focus T cell antigen exploration could afford substantial advantages for discovering and monitoring the anti-cancer immune responses of patients enrolled on clinical trials.

**Methods:**

We vaccinated female BALB/c mice with a novel combination of an autophagosome-enriched vaccine derived from 4T1 mammary carcinoma along with poly-I:C adjuvant, then screened serum for IgG binding to arrays of 15mer peptides containing known mutation sites in 4T1. Simultaneously, we primed CD8+ T cell cultures from these same animals with 8-11mer peptides derived from these antigens. These primed T cells were then stimulated to measure recognition of the peptides or live 4T1 cells by IFNγ release.

**Results:**

Vaccinated animals demonstrate increases in antigen-specific CD8+ T cell recognition of 4T1 tumor cells and peptides. For proteins confirmed in 4T1 cells and vaccine by mass spectrometry, there is a correlation between this increased CD8+ T cell IFNγ release and serum IgG binding to individual peptide antigens.

**Conclusions:**

These results suggest it is possible to observe some features of a patient’s antigen-specific T cell repertoire via an antibody surrogate, which has implications for tumor antigen discovery and clinical monitoring of antigen-specific anti-tumor immunity.

**Electronic supplementary material:**

The online version of this article (10.1186/s40425-018-0331-0) contains supplementary material, which is available to authorized users.

## Background

A large background of autoantibody signals to thousands of normal human proteins is frequently observed in IgG biomarker surveys [1–5]. On average, over 20% of the entire surveyed human proteome is targeted by a unique landscape of these autoantibodies in healthy individuals [1]. Such preexisting or “natural” antibody landscapes are thought to be the result of prior adaptive immunity to similar peptide mimics found in commensal microbes, foods, environmental exposures, infections, and autologous proteins. In spite of the frequency of autoantibodies observed in humans, and the similarity between many types of tumor antigens and autologous targets, it is not known whether these serum antibodies or changes in their abundance might also hint at the antigen-specific behavior of an individual’s T cell repertoire. Others have used antibody as a surrogate measure of antigen-specific anti-tumor immunity [4–6], and we hypothesized that IgG antibody signals would be more likely to overlap with features of antigen-specific CD8+ T cell recognition than expected by chance. Potential mechanisms for such a relationship could occur via overlap with the underlying CD4+ T cell repertoire necessary for activating both CD8+ T cells and B cells, or from antibody-aided T cell activation via Fc receptor targeting of antigens to antigen presenting cells. Improved understanding of the antigen-specific relationships between antibody and T cell responses to tumor antigens could lead to improved immune monitoring for cancer patients and a deeper understanding of what features define clinically-relevant tumor antigens.

Although the overall benefit of B cell responses to cancer remains controversial [7–9], there is a long history of surveys for antigen-specific anti-tumor antibodies [10, 11]. One type of anti-tumor immunity increasingly recognized as important to improved outcomes for patients with cancer are responses to tumor-specific single nucleotide variant (SNV) neoantigens [12–16], which differ by a single amino acid from their wild-type (WT) counterpart autoantigens. We sought to screen for IgG antibodies to peptides centered at previously reported mutation sites in the 4T1 tumor model in both SNV neoantigen and their WT autoantigen counterpart versions. We hypothesized that patterns in these antibody profiles would relate to the 4T1 vaccine-induced T cell recognition of those same mutation sites. In viral immunity, there are documented examples of IgG antibody responses mirroring CD4+ responses at the level of individual antigens [17–19]. Similar to the viral literature, potential links have been observed between anti-tumor antibodies and T cell responses to specific tumor antigens [20, 21], and increased antigen-specific antibody responses have been observed in association with improved outcomes following immunotherapy treatments typically understood to depend on T cells [4, 6, 22].

## Methods

### Study design

For this work, we chose 4T1, a metastatic murine mammary carcinoma model in BALB/c mice with a limited number of previously described neoantigens, and a vaccine that is known to both work in 4T1 therapeutically and generate cross-reactive immunity to diverse unrelated tumors [23, 24]. This model provided an opportunity to repeatedly interrogate antigen-specific immune responses to specific components of our vaccine in a well-controlled system. The 4T1 tumor cell line was a gift of Emmanuel Akporiaye (Earle A. Chiles Research Institute, Portland, OR), from stocks received from Suzanne Ostrand-Rosenberg (UMBC, Baltimore, MD). Cell line identity was confirmed identical to ATCC 4T1 and free from *Mycoplasma* and other common eukaryotic contaminants via microsatellite profiling (IDEXX RADIL). Tumor cells were thawed directly from the confirmed bank and passaged less than 4 times before use. Cells were cultured in complete media consisting of RPMI-1640 (Lonza) with 1% L-Glutamine (Lonza), 1% Sodium Pyruvate (Lonza), 1% Non-essential Amino Acids (Lonza), 0.1% Beta Mercaptoethanol, 50 mg/L Gentimicine Sulfate, and 10% fetal bovine serum (Atlas Biologicals Lot # 1070612). Production of three 4T1 autophagosome-enriched vaccine lots was performed as previously described [23, 25]*.* In brief, tumor cells were seeded into T225 flasks, grown to ~ 70% confluence, and treated with 20 mM ammonium chloride and 100 nM Bortezomib (Velcade) to induce autophagosome formation. Treated 4T1 cells were harvested and sonicated to release autophagasomes. Suspended autophagasomes were harvested with centrifugation at 12,000 G. Protein content was measured by a BCA assay using bovine serum albumin as a standard, and harvested 4T1 autophagosome-enriched vaccine was diluted to a protein concentration of 1 mg/mL in hetastarch vehicle and frozen at − 80 °C until use. Age-matched 14–20 week old female BALB/c mice (Jackson Laboratories) were vaccinated in both inguinal nodes with a total of 10 μg 4T1 autophagosome-enriched vaccine plus 3 μg of Vaccigrade poly-I:C (Sigma-Aldrich) in 20 μL hetastarch carrier, vaccine and carrier alone, poly-I:C adjuvant and carrier alone, or left untreated. Animals were boosted after two weeks with a single subcutaneous injection of the same total dose in the left flank. After another two weeks serum was harvested for analysis or mice were challenged with 5000 live 4T1 cells in the left mammary fat pad. Tumor growth in challenged mice was measured thrice weekly for 30 days until immunohistochemistry and tumor bearing serum experiments, or until a maximal area of 150 mm^2^, which was the determinant for death in overall survival experiments.

### Multispectral IHC

Day 30 4T1 tumors were pretreated for 24 h in a zinc solution, placed in 70% ethanol, and then paraffin embedded until staining as previously described [26]. Five μm sections were cut and fluorescently stained with DAPI and specific antibodies to CD8a (53–6.7, BD Pharmingen), F4/80 (Cl:A3–1, Bio Rad), CD3 (SP7, Spring Bioscience), FOXP3 (FJK-16 s, eBioscience), and CD4 (RM4–5, BD Biosciences) via tyramide signal amplification. Multispectral fields were imaged with a multispectral microscope (PerkinElmer, Vectra) and 15 representative 20× fields per sample were quantified with vendor software (PerkinElmer, Inform).

### 4T1 whole exome sequencing and variant detection

DNA was then isolated from our 4T1 cell line bank using a Qiagen DNeasy kit and sent to a contractor for whole-exome sequencing (Otogenetics) at a target 50× coverage depth. Using CLC Genomics Workbench v7.04, the resulting Illumina FASTQ files were aligned to the mm10 reference genome using CLC NGS core tools, a BWS algorithm, to preserve annotations. Known SNVs and indels in BALB/cJ versus mm10 were subtracted using a variant file downloaded from the Sanger mouse genome project (www.sanger.ac.uk/science/data/mouse-genomes-project). Heterozygous non-synonymous protein-coding variants detected > 10 times were determined to be 4T1-specific SNV mutation candidates.

### 4T1 15mer mutation site peptide arrays

Mutation site candidates identified from our sequencing were compared to a list of heterozygous nonsynonymous protein coding 4T1 SNVs identified in prior publications [27, 28]. One of these studies reported immunologic response data to 17 4T1 neoantigens [27], and we included all of these previously reported immunogenic 4T1 mutation sites on the arrays. As space allowed in the array design, we additionally included 66 of the 81 total mutation sites identified by both our independent sequencing and confirmed by at least one of the other reports. The Mouse ENSMBL protein database was downloaded from BioMart (www.ensembl.org/biomart) [29], and 15mer wild-type peptide sequences were extracted centered at the 75 selected coordinates. The 15mer wild-type sequences were then altered to the identified SNV versions for a total of 150 WT and SNV peptides. These 150 peptides were printed in triplicate on 60 replicate custom peptide arrays along with the known 4T1 retroviral antigen AH1 [30] and anti-mouse IgG control spots by JPT Peptides (Berlin, Germany). Twenty of these arrays were used in preliminary experiments, and forty in follow-up experiments paired with T cell data. Whole mouse sera were pooled from 2 to 3 animals per experimental group, diluted 1:200, and incubated on the peptide arrays for one hour at 30 °C. IgG signals were detected with a fluorescent anti-mouse IgG secondary. All samples reacted to anti-mouse IgG control spots. Each array spot was imaged with a high resolution fluorescence scanner and its intensity quantified with GenePix spot-recognition software (Molecular Devices). Resulting IgG fluorescence intensity values were averaged across each of the three replicate spots for further analysis. In preliminary studies, the average intensity values from all initial 20 arrays were normalized simultaneously using an interquartile range transformation performed using BRB-ArrayTools v4.5.0 developed by Dr. Richard Simon and the BRB-Array Tools Development Team (brb.nci.nih.gov/BRB-ArrayTools). These preliminary arrays are presented in Additional file 1: Data file S1, and were used for selecting antigens to additionally investigate in T cell assays. The 40 follow-up arrays paired with T cell data were analyzed as the raw average of the three replicate spots without such normalization, are plotted in figures, and additionally included in Additional file 1: Data file S1.

### In vitro T cell IFN ***γ*** release peptide assays

Antigens selected for additional profiling via IFNγ T cell assays were selected based on a profile of the preliminary peptide array data. We selected thirty-one antigens that spanned a range of properties: sites with a strong preexisting IgG background signal, sites with a post-vaccine IgG signal increase across multiple experiments, sites with high and low predicted MHCI affinity, and mutation sites without any of these distinctions but previously reported as immunogenic [27]. All experiments were performed using pooled splenocytes from 2 to 3 individual female BALB/c mice harvested two weeks after their second vaccination. In the case of CD8+ enriched experiments, CD4+ cells were depleted in vivo three days prior to spleen harvest using 200 μg of GK1.5 anti-CD4 antibody administered IP. CD4 depletion was confirmed by flow cytometry. After ACK lysis of red blood cells, 1 × 10^6^ splenocytes were plated into each well of 96 well round-bottom tissue culture plates and given primary stimulation in complete media with 10% FBS and 5 μM of either SNV or WT versions of mutation site peptides manufactured by A&A Labs (San Diego, California). 15mer peptides matched the IgG arrays, and 8-11mer minimal peptides designs were based on predicted ability to bind MHCI. Both WT and SNV 8-11mer peptides were based on the length and frame of the top predicted MHCI binding minimal 8-11mer SNV peptide identified using NetMHCpan v2.8 Server. NetMHCpan, was used to calculate predicted H2-Kd, H2-Dd, and H2-Ld MHCI binding scores for all possible WT and SNV 8mers, 9mers, 10mers, and 11mers that include the mutation site [31]. After 48 h of primary peptide stimulation, IL2 was added at 10 Cetus units/mL. After an additional 96 h, contents of each well were washed and split onto either 2° peptide restimulation with 5 × 10^5^ irradiated splenocytes, irradiated splenocytes alone, media only, or plated onto 1 × 10^5^ live 4T1 cells. Supernatants were harvested after an additional 20 h, frozen at − 80 °C, and later analyzed for IFNγ by ELISA.

### TMT LC-MS/MS of 4T1 cells and autophagosome-enriched vaccine

Quantitative tandem mass tag (TMT) liquid chromatography tandem mass spectrometry (LC-MS/MS) was performed by the Proteomics Shared Resource at Oregon Health & Science University on three 4T1 autophagosome-enriched vaccine lots and three paired samples of untreated whole 4T1 cells. Samples were lysed using a probe sonicator and protein concentration was estimated using BCA assay. Forty μg of protein per sample was trypsin digested in solution. In brief, samples were dried, dissolved in 10 μL of 4X buffer (8 M urea,1 M Tris (pH 8.5), 8 mM CaCl_2_, 0.2 M methylamine), reduced, alkylated, diluted to a final 2 M urea concentration and digested by addition of 1.6 μg of sequencing grade trypsin overnight (ProMega) Completion of the digestion was confirmed by 1-D gel analysis. Twenty-five μg of each digested sample was then solid phase extracted using Oasis HLB 1cm^3^ cartridges (Waters Corporation), and peptides dried by vacuum centrifugation. Samples were labeled with 10-plex TMT reagents (Thermo Scientific), pooled together, and on-line two dimensional reverse phase/reverse phase (RP-RP) liquid chromatography used to separate into 9 fractions at high pH, and each fraction further separated at low pH. Peptides were analyzed using an Orbitrap Fusion Mass Spectrometer (Thermo Scientific) with a synchronous precursor selection MS3 TMT method [32]. Twenty μL samples (32.9 μg) were injected onto a NanoEase 5 μM XBridge BEH130 C18 300 μM × 50 mm column (Waters) at 3 μL/min in a mobile phase containing 10 mM ammonium formate (pH 10). Peptides were eluted by sequential injection of 20 μL volumes of 14, 20, 22, 24, 26, 28, 30, 40, and 90% acetonitrile (ACN) in 10 mM ammonium formate (pH 10) at a 3 μL/min flow rate. Eluted peptides were diluted with mobile phase containing 0.1% formic acid at a 24 ul/min flow rate and delivered to an Acclaim PepMap 100 μM × 2 cm NanoViper C18, 5 μM trap (Thermo Scientific) on a switching valve. After 10 min of loading, the trap column was switched on-line to a PepMap RSLC C18, 2 μM, 75 μM × 25 cm EasySpray column (Thermo Scientific). Peptides were then separated at low pH in the 2nd dimension using a 7.5–30% ACN gradient in mobile phase containing 0.1% formic acid at 300 nL/min flow rate. Each 2nd dimension LC run required 2 h for separation and re-equilibration, so the entire LC/MS method required 18 h for completion. Survey scans were performed in the Orbitrap mass analyzer (resolution = 120,000), and data-dependent MS2 scans performed in the linear ion trap using collision-induced dissociation (normalized collision energy = 35) following isolation with the instrument’s quadrupole. Reporter ion detection was performed in the Orbitrap mass analyzer (resolution = 60,000) using MS3 scans following synchronous precursor isolation of the 10 most intense ions in the linear ion trap, and higher-energy collisional dissociation in the ion-routing multipole (normalized collision energy = 65).

Mass spectrometry data was processed against the UniProt Swiss-Prot canonical mouse protein database (v. 2014_05, 16,669 sequences) with SEQUEST HT in Proteome Discoverer v1.4 (Thermo Scientific). Search settings were: monoisotopic parent ion mass tolerance of 1.25 Da, monoisotopic fragment ion tolerance of 1.0 Da, tryptic cleavage with up to 2 missed cleavages, variable modification of oxidized methionine, and static modifications for TMT reagents (peptide N-term and lysines) and alkylated cysteines. Peptide sequence assignments were validated using Percolator [33] q-values (less than 0.05) and 20 ppm delta mass agreement between measured and theoretical peptide masses. TMT reporter ion intensities of individual peptides were exported as text files and processed with in-house scripts. A median reporter ion intensity cutoff of 1500 was used to reject low quality peptides, and all reporter ion intensities for unique peptides matched to each respective protein were summed to create total protein intensities. A minimum of 2 peptides contributing to the protein total was required for each identification to improve data quality. Protein identification, quantitative information, and additional UniProt annotations were tabulated for all proteins and are listed in Additional file 1: Data file S1. A total of 4416 proteins were identified and quantification was done on 4196 proteins (excluding contaminants). Only this discovery confirmation, and not quantitative abundance, was used to separate experimental groups in Fig. 6, and Additional file 2: Figures S5–7.

### Statistical analyses

Analyses were performed on either summary data or individualized experiments, and this information is placed alongside the specific type of test performed and *p*-value (P) within the figure legends. All statistical tests were considered significant at the *P* < 0.05 level and were performed with Prism 7 (GraphPad). In general, parametric comparisons were either two sample t-tests or paired t-tests, and non-parametric tests were Wilcoxon matched-pairs signed rank tests. Significance of all correlations was determined by linear regression and Pearson correlation coefficient. Graphs and statistics in Figs. 2, 3, 4, 5, and 6 and Additional file 2: Figures S3–7 can be recreated from values presented in Additional file 1: Data file S1.

## Results

**Fig. 1:**
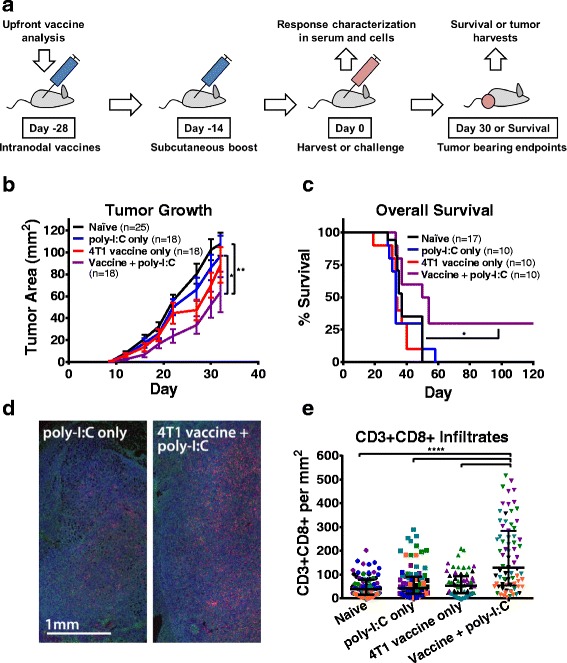
Prophylactic autophagosome vaccination delayed 4T1 tumor growth, improved overall survival, and increased intratumoral CD3 + CD8+ infiltration. **a** Mice were vaccinated in both inguinal lymph nodes with 4T1 autophagosome-enriched vaccine plus poly-I:C, vaccine alone, adjuvant alone, or left untreated. Animals were boosted subcutaneously after two weeks. After another two weeks, sera or spleens were harvested at Day 0 for in vitro antibody and T cell assays or animals were challenged with live 4T1 tumor cells for survival endpoints, tumor bearing sera, and immunohistochemistry. **b** Upon challenge, reduced average tumor growth was observed in combination vaccine + poly-I:C pretreated animals with maximum separation occurring at Day 22 versus poly-I:C alone (*P* = 0.04) and Day 27 versus naïve animals (*P* = 0.002) by Dunnett’s multiple comparisons test. Data were pooled from five independent experiments with error bars plotted as the standard error of the mean. **c** Overall survival was improved in combination treatment versus all other groups (*P* = 0.02) by Gehan-Breslow-Wilcoxon test. Data were pooled from three independent experiments. **d-e** Zinc and alcohol fixed day 30 4T1 tumors were stained for six color immunohistochemistry with tyramide signal amplification. **d** Three color representative image showing CD8+ (red), F4/80 (green), and DAPI (blue). **e** Fifteen 20× fields were imaged for each of 4 to 6 tumors per group and quantified for CD3 + CD8+ per mm^2^ (fields from individual tumors colored separately). Higher numbers of CD3 + CD8+ infiltrates were seen in the fields from vaccine + poly-I:C pretreated tumors versus all other groups (*P* < 0.0001) by t-test. Lines plotted are the median and interquartile range

We have previously demonstrated the benefits of our tumor cell-derived autophagosome-enriched vaccine model [23–25, 34], a vaccine that has demonstrated both prophylactic and therapeutic efficacy against both syngeneic and unrelated tumors. This vaccine platform is currently in clinical trials, and has demonstrated increased therapeutic efficacy when combined with anti-OX40 for the treatment of established 4T1 [24], a metastatic mammary carcinoma model with established sequencing and neoantigen immunity data [27, 28, 35]. In order to evaluate the immune response induced by this vaccine, independent of the effect of a progressively growing tumor, naïve tumor-free BALB/c mice were studied. Animals received a 4T1 autophagosome-enriched vaccine + poly-I:C adjuvant injected into the inguinal lymph nodes of naïve female BALB/c mice. A single booster vaccination was given subcutaneously at 2 weeks, and, at 4 weeks, animals were killed for sera harvests or challenged with live 4T1 tumor cells (Fig. 1a). Challenged animals that had received prophylactic 4T1 autophagosome-enriched vaccine + poly-I:C, but not either alone, benefitted from a significant delay in tumor growth (Fig. 1b), results similar to our prior publications. Additionally, the only group that demonstrated a statistically significant increase in long-term survival was the combination treatment (Fig. 1c). It should be noted that while the level of protection is small, 4T1 is considered to be a poorly immunogenic tumor as vaccination with irradiated 4T1 tumor cells fails to protect any animals from a tumor challenge [36]. Additionally, when this 4T1 autophagosome vaccine is combined with a T cell agonist, anti-OX40, therapeutic efficacy is significantly increased [24]. We next sought to determine whether there were any differences in intratumoral T cell infiltrates in these tumors by immunohistochemistry that would help explain the treatment benefit; numerous studies have linked increased T cell infiltrate with improved outcomes following original work by Galon and colleagues [37]. We stained sections from day 30 4T1 tumors as previously reported [26] for CD3, CD4, CD8, FOXP3, and F4/80 and quantified the infiltrates. Versus all other groups, including adjuvant-only controls, combination vaccinated animals demonstrated an increase in CD3 + CD8+ infiltrates (Fig. 1d, e). Versus adjuvant-only controls, these same tumors demonstrated no difference in CD3 + CD4 + FOXP3- or CD3 + CD4 + FOXP3+ infiltrates (Additional file 2: Figure S1A, B). These results demonstrate that our combination autophagosome-enriched vaccine creates increased frequencies of CD8+ T cells that are capable of trafficking to 4T1 tumors in vivo. These results correlate with delayed in vivo tumor growth similar to previous clinical reports [37].

We next sought to determine whether IgG antibodies could be used to indicate immunity to known mutation sites in 4T1 after treatment with our vaccine model. We were led to this by others whose observations have suggested links between IgG antibody and T cell responses to human tumor antigens [6, 21]. We isolated DNA and performed whole-exome sequencing on our 4T1 cell bank, and used the sequencing data to identify heterozygous single nucleotide variants by comparison to a female BALB/cJ reference sequence; subsequently referred to as SNVs. We used SNVs that were both identified in previous reports [27, 28, 35] and confirmed by our sequencing to design a custom 15mer peptide array for 75 SNV neoantigens and 75 alternate allele wild type (WT) autoantigens centered at 4T1 mutation sites, as well as the known retroviral antigen AH1 [30] (Additional file 2: Figure S2). A number of these mutation sites have been previously reported as immunogenic to murine CD4+ or CD8+ T cells [27]. Each array contained all peptides printed in triplicate along with anti-mouse IgG controls. We ran preliminary IgG arrays with sera harvested from animals used in the tumor challenge and in vitro experiments already presented (Fig. 1a-e), and these preliminary arrays were used to identify outlier antigens of interest for final IgG array experiments paired with IFNγ T cell assays. Data from both preliminary IgG arrays and final IgG arrays paired with T cell IFNγ release data are available (Additional file 1: Data file S1).

**Fig. 2:**
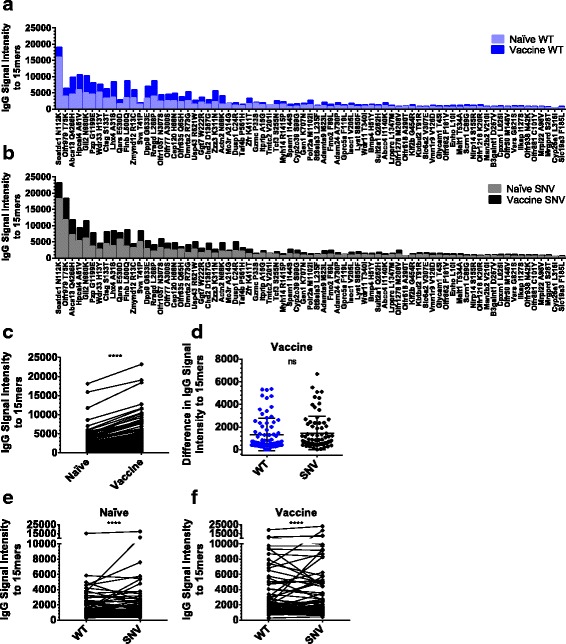
Vaccinated animal sera had increased IgG antibodies to peptides centered at mutation sites in 4T1. **a-f** Data are from five independent pairs of IgG arrays reacted with pooled naïve or vaccinated mouse serum. Each array consists of 151 15mer peptides printed in triplicate and centered at WT autoantigen and SNV neoantigen mutation-sites in 4T1. **a** Average serum IgG fluorescence signal intensity versus 15mer peptides centered at WT versions of listed 4T1 mutation-sites in naïve and vaccinated animals sorted by the combined WT and SNV IgG signals observed in naïve animals. **b** Average serum IgG fluorescence signal intensity versus 15mer peptides centered at SNV versions of listed 4T1 mutation-sites in naïve and vaccinated animals sorted by the combined WT and SNV IgG signal observed in naïve animals. **c-d** Data are plotted as average values, but statistics are computed from all individualized pairs of experimental values. **c** Vaccinated animals demonstrated increased serum IgG signal intensity to a WT and SNV 15mer peptides (*P* < 0.0001) by Wilcoxon matched-pairs signed rank test, (**d**) but these observed increases in IgG signal intensity from vaccine groups were not significantly higher for SNV peptides than WT peptides (*P* = 0.26) by Wilcoxon matched-pairs signed rank test. However, there are stronger IgG signal intensities for SNV neoantigens than paired WT autoantigens in serum from both (**e**) naïve animals (*P* < 0.0001), and (**f**) vaccinated animals (*P* < 0.0001) by Wilcoxon matched-pairs signed rank test

The serum array data demonstrate IgG binding signals against 4T1 peptides from both naïve and vaccinated animal sera, with increased average IgG signals in vaccine groups against many individual WT autoantigen 15mer peptides (Fig. 2a), and SNV neoantigen 15mer peptides (Fig. 2b). The IgG signals to both WT and SNV 4T1 peptides were significantly higher in sera from vaccinated animals (Fig. 2c), but these increased IgG signals after vaccination did not significantly favor SNV neoantigen over WT autoantigen peptides (Fig. 2d). However, there were stronger overall IgG signals against SNV peptides in serum from both naïve (Fig. 2e) and vaccinated (Fig. 2f) animals, suggesting a background landscape of preexisting serum antibodies that favors neoantigens over autoantigens.

Prior to the final IgG array experiments presented in Fig. 2, we had used preliminary IgG arrays to identify a number of 4T1 antigens which had strong increases in IgG signals after vaccination or strong preexisting IgG signals in naïve animals. We used these results to select a smaller set of antigens for investigation in parallel T cell assays, and ran these T cell assays with splenocytes from the same animals whose serum IgG was analyzed in Fig. 2. These T cells were stimulated with either 15mer peptides matching the IgG array or predicted MHCI binding 8-11mer peptide designed with MHCI prediction software [31]. The chosen mutation sites are listed in relation to IgG array data in Additional file 1: Data file S1, and were tested as both WT autoantigen and SNV neoantigen peptides.

**Fig. 3:**
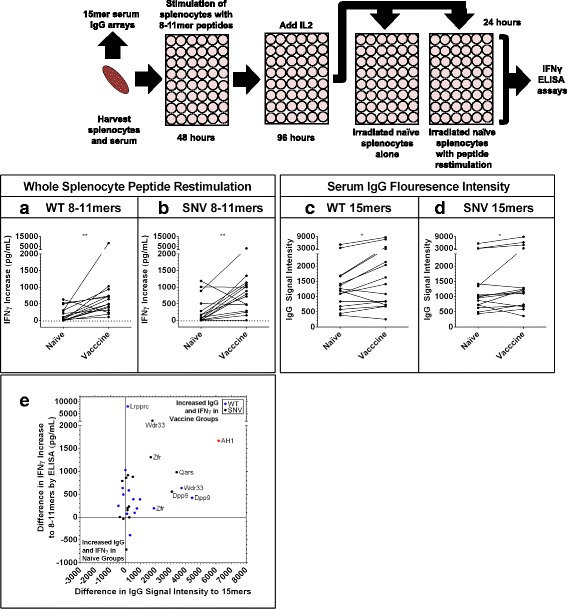
Simultaneous increases in IgG signals to 15mers and splenocyte IFNγ recognition of individual 8-11mer antigens. Serum and splenocytes were harvested from naïve and 4T1 autophagosome-enriched vaccine + poly-I:C vaccinated animals. Serum was run on the 15mer arrays presented previously (Fig. 2). Splenocytes were stimulated with WT and SNV versions of top predicted MHCI binding 8-11mer mutation site peptides for 48 h, then expanded on IL2 for an additional 96 h before wells were split and restimulated with either naïve splenocytes or naïve splenocytes pulsed with a second stimulation of peptide. Graphs are of the average increase in IFNγ secretion by ELISA in wells with peptide restimulation over splenocytes alone for *n* = 3 experiments with vaccine groups and *n* = 2 experiments with naïve groups. **a**, **b** Increase in average IFNγ secretion in vaccine groups upon secondary exposure to *n* = 15 different WT (*P* = 0.002) (**a**) and *n* = 15 different SNV (*P* = 0.005) peptides (**b**) by Wilcoxon matched-pairs signed rank test. **c**, **d** Simultaneous serum IgG array recognition data for 15mer peptides centered at these same mutation sites from the same *n* = 3 vaccine groups and *n* = 2 naïve groups used in splenocyte assays. Increase in average IgG signal intensity to *n* = 15 different WT (*P* = 0.01) (**c**) and *n* = 15 different SNV (*P* = 0.02) (**d**) peptides by Wilcoxon matched-pairs signed rank test. **e** Combined data previously presented in (**a-d**) plots average differences in IgG and IFNγ recognition for each of the *n* = 15 WT and *n* = 15 SNV mutation sites along with AH1. Positive values represent increased signals in vaccine groups and negative values represent increased signals in naïve groups. Values in upper-right quadrant demonstrated simultaneous increases in IgG and splenocyte IFNγ recognition of individual 4T1 mutation-site antigens in vaccine groups. However, there was no significant overall correlation of these increases in recognition (*P* = 0.6) by Pearson correlation coefficient

T cells from vaccinated animals have increased recognition of both WT and SNV 8-11mer 4T1 peptides (Fig. 3a-b), and serum from these vaccinated animals also demonstrates increased IgG binding to 15mer peptides containing this same group of mutation sites (Fig. 3c-d). For several of these 4T1 antigens, we observed simultaneous increases in IgG 15mer signal intensity and T cell recognition of 8-11mer peptides for specific antigens in vaccinated animals (Fig. 3e). A similar result was observed for splenocyte assays involving WT 15mer peptides (Additional file 2: Figure S3A-E), but naïve splenocytes were additionally able to recognize SNV 15mer peptides after our culture process. Because of this increased naïve recognition we did no further experiments with 15mer peptides.

**Fig. 4:**
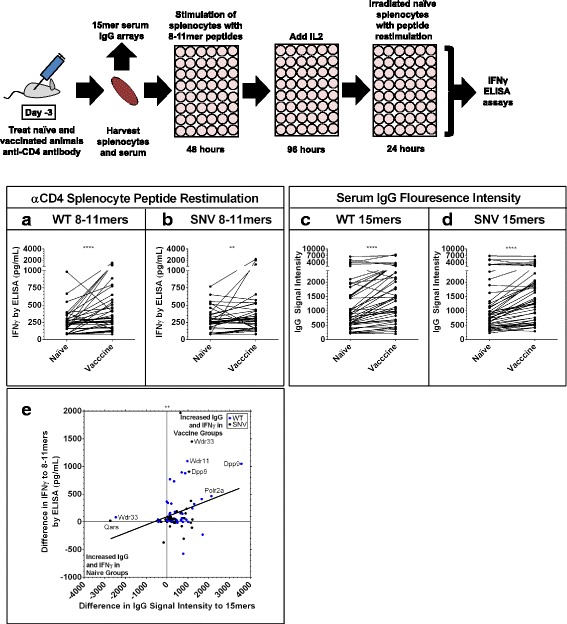
Simultaneous increases in IgG signals to 15mers and CD8+ IFNγ recognition of individual 8-11mer antigens. Serum and CD4-depleted splenocytes were harvested from naïve and 4T1 autophagosome-enriched vaccine + poly-I:C vaccinated animals. Serum was run on the 15mer arrays presented previously (Fig. 2). CD4-depleted splenocytes were stimulated with WT and SNV versions of top predicted MHCI binding 8-11mer mutation site peptides for 48 h, then expanded on IL2 for an additional 96 h before wells were split and restimulated with naïve splenocytes pulsed with a second stimulation of peptide. Graphs are of the IFNγ secretion for each individual paired experiment from *n* = 3 paired replicates with vaccine and naïve groups, each involving *n* = 15 different WT and *n* = 15 SNV peptide experiments. **a, b** Increase in IFNγ secretion in vaccine groups upon secondary exposure to *n* = 45 paired experiments with WT peptides (*P* < 0.0001) (**a**) and *n* = 45 paired experiments with SNV (*P* = 0.005) peptides (**b**) by Wilcoxon matched-pairs signed rank test. **c**, **d** Simultaneous serum IgG array recognition data for 15mer peptides centered at these same mutation sites from the same *n* = 3 vaccinated animal groups and *n* = 3 naïve groups used in splenocyte assays. Increase in average IgG signal intensity to *n* = 45 paired WT peptide experiments (*P* < 0.0001) (**c**) and *n* = 45 SNV peptide experiments (*P* < 0.0001) (**d**) by Wilcoxon matched-pairs signed rank test. **e** Combined data previously presented in (**a-d**) plots average differences in IgG and IFNγ recognition for each of the *n* = 45 WT experiments and *n* = 45 SNV experiments. Positive values represent increased signals in vaccine groups and negative values represent increased signals in naïve groups. Values in upper-right quadrant demonstrated simultaneous increases in IgG and splenocyte IFNγ recognition of individual 4T1 mutation-site antigens in vaccine groups. There was a significant overall correlation of these increases in IgG and CD8+ IFNγ recognition (*P* = 0.0039) by Pearson correlation coefficient

Since we observed increased recognition of 8-11mer 4T1 peptides by T cells from vaccinated animals, and initial immunohistochemistry experiments suggesting a greater role for CD3 + CD8+ cells than CD3 + CD4+ cells for tumor control in this model (Fig. 1 d,e; Additional file 2: Figure S1A,B), we next sought to confirm the role of CD8+ T cells with an enriched population of CD8+ T cells. Experiments were performed as before except with the addition of a CD4-depleting antibody in vivo prior to spleen harvest. Compared to naïve animals, vaccinated animals demonstrated stronger CD8+ T cell IFNγ recognition of both WT autoantigen and SNV neoantigen 8-11mer peptides from 4T1 (Fig. 4a,b). Interestingly, Serum from these vaccinated animals also demonstrated a significantly (*p* < 0.0001) increased IgG binding to 15mer WT peptides as well as 15mer SNV peptides containing these mutation sites (Fig. 4c,d), and there was a significant (*p* = 0.0039) correlation between increased IgG binding to 15mer peptides after vaccination and increased IFNγ recognition of both the WT and SNV 8-11mer peptides by CD8+ T cells. This suggests that at least in some cases, vaccination with an autophagosome vaccine leads to the development of a coordinated immune response that results in CD8+ T cell antigen recognition in tandem with increased IgG antibody recognition of those same tumor peptides.

**Fig. 5:**
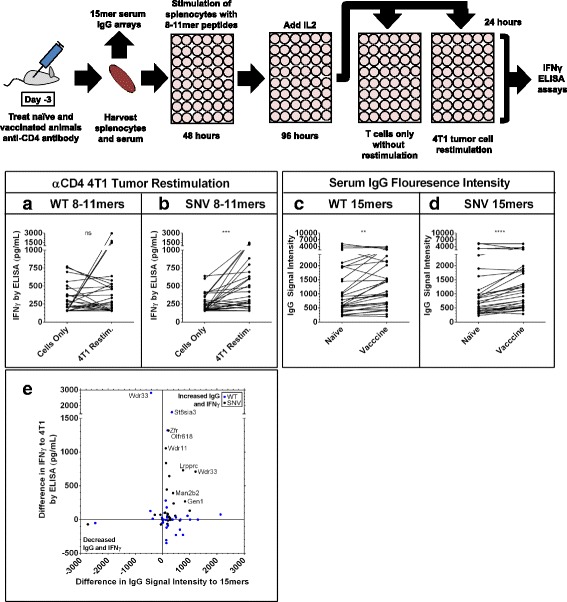
Simultaneous increases in IgG signals to 15mers and improvements in CD8+ IFNγ recognition of tumor. Serum and CD4-depleted splenocytes were harvested from 4T1 autophagosome-enriched vaccine + poly-I:C vaccinated animals. Serum was run on the 15mer arrays presented previously (Fig. 2). CD4-depleted splenocytes were stimulated as presented previously (Fig. 4), then placed in empty wells or restimulated with 4T1 tumor cells. Graphs are of the IFNγ secretion for each individual paired experiment from *n* = 2 paired replicates with CD8+ T cells only or CD8+ T cells plus live 4T1 cells, each pair initially stimulated with one of *n* = 15 WT or *n* = 15 SNV peptides. **a**, **b** IFNγ secretion in 4T1 tumor restimulated groups demonstrated outliers, but no overall increased 4T1 recognition after primary exposure to *n* = 30 paired experiments with WT peptides (*P* = 0.65) (**a**), but did show overall increased 4T1 recognition after primary exposure to *n* = 30 paired experiments with SNV peptides (*P* = 0.0002) (**b**) by Wilcoxon matched-pairs signed rank test. **c**, **d** Simultaneous serum IgG array recognition data for 15mer peptides centered at these same mutation sites from the same *n* = 2 vaccinated animal groups used in splenocyte assays and *n* = 2 naïve group controls. Increase in average IgG signal intensity to *n* = 30 paired WT peptide experiments (*P* < 0.0022) (**c**) and *n* = 30 SNV peptide experiments (*P* < 0.0001) (**d**) by Wilcoxon matched-pairs signed rank test. **e** Combined data previously presented in (**a-d**) plots average differences in IgG and IFNγ recognition for each of the *n* = 30 WT experiments and *n* = 30 SNV experiments. Positive values represent increased IgG signals versus naïve controls and increased IFNγ recognition of 4T1 tumor over T cells only. Values in upper-right quadrant demonstrated increases in serum IgG recognition of that antigen, and a simultaneous ability for that antigen to improve CD8+ T cell IFNγ recognition of live 4T1 cells. However, there was no significant direct correlation of these increases in IgG and CD8+ IFNγ recognition (*P* = 0.95) by Pearson correlation coefficient

In order to determine the relevance of these results to tumor recognition, we performed two of the CD4-depleted experiments with additional restimulation groups of live 4T1 tumor cells. In vitro priming of CD8+ T cells from vaccinated animals with 8-11mer WT autoantigen peptides produced tumor recognition in three cases, but not as an overall group (Fig. 5a). In contrast, a larger fraction of CD8+ T cells from vaccinated animals stimulated with 8-11mer SNV neoantigen peptides demonstrated improved 4T1 tumor recognition (Fig. 5b). It is interesting to note that when evaluating the development of strong IFN-γ responses, arbitrarily set at 1000 pg, WT peptides induced 3 strong responses and the SNV peptides induced 4 strong responses. Similar to previous results, this coincided with increased IgG antibody to both WT and SNV 15mer versions of these mutation sites (Fig. 5c,d), which often resulted in simultaneous improvements in IgG and tumor recognition related to specific 4T1 SNV neoantigens (Fig. 5e). Though not directly correlative for the whole group of candidate 4T1 antigens, there were several post-vaccine IgG signal increases which matched the antigens that improved CD8+ T cell responses against 4T1 tumor.

Our IgG array data demonstrated that many of the strongest vaccine-induced antibody responses to 4T1 peptides could be detected, albeit at lower levels, in naive animals (Additional File 2: Fig. S4).

**Fig. 6:**
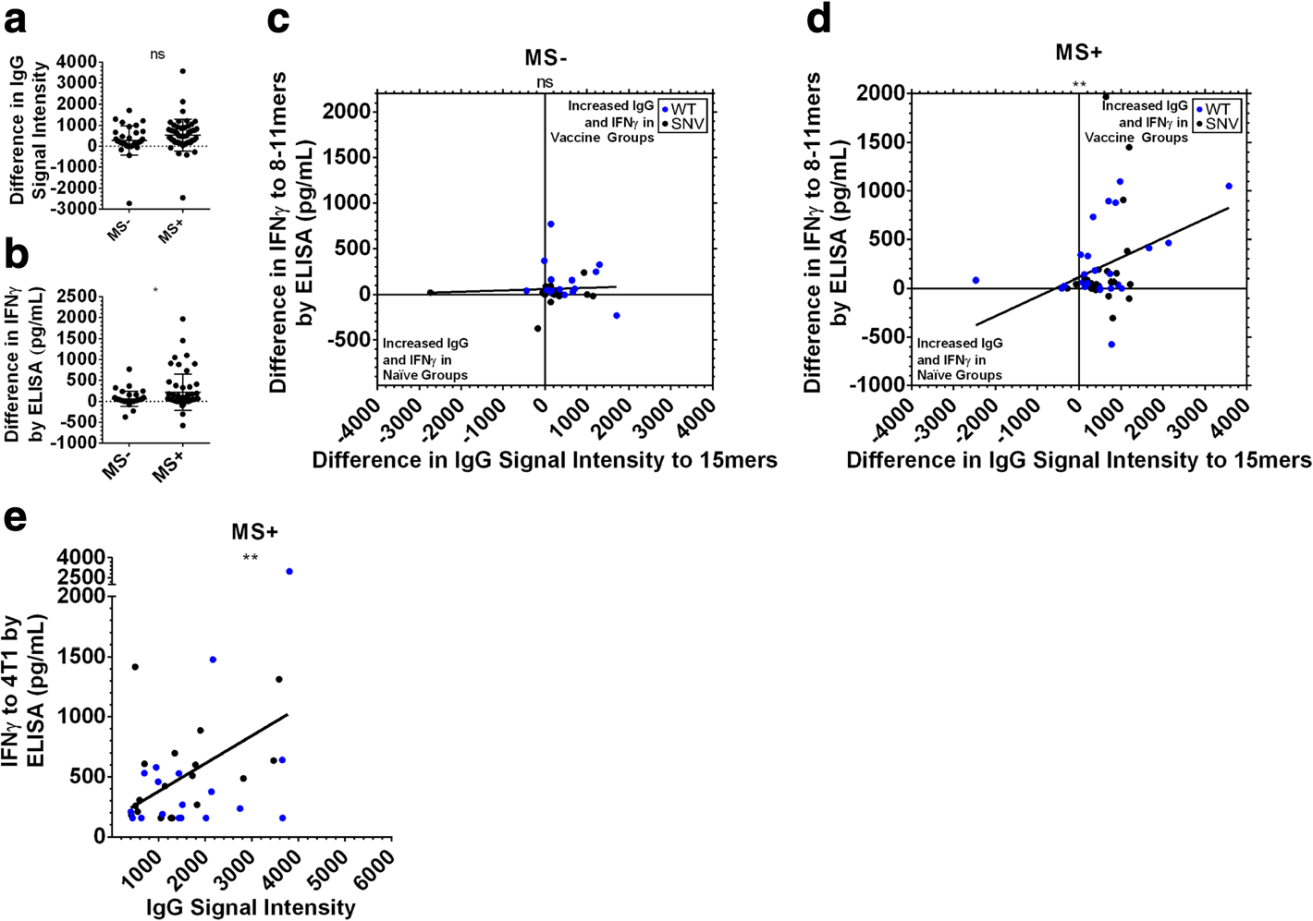
Simultaneous vaccine-induced IgG and CD8+ IFNγ recognition of 4T1 tumor and peptides confirmed by LC-MS/MS. LC-MS/MS was performed to determine whether proteins containing the 4T1 peptides were present in live 4T1 cells and 4T1 autophagosome-enriched vaccine. Of the 15 4T1 WT and SNV pairs additionally analyzed in T cell assays, there were *n* = 9 WT and SNV antigen pairs from proteins confirmed in 4T1 by mass spectrometry and *n* = 6 WT and SNV antigen pairs not confirmed by mass spectrometry. **a-d** Data shown are from experiments previously presented in Fig. 4. Vaccinated animals demonstrated increased serum IgG to the peptides analyzed in T cell assays; however, there was no improved IgG response (**a**) to the WT and SNV peptides confirmed by mass spectrometry over unconfirmed proteins (*P* = 0.1) by unpaired t-test. However, confirmed presence of the antigenic protein in the vaccine by mass spectrometry (**b**) resulted in improved IFNγ secretion in vaccine groups upon secondary exposure to WT and SNV 4T1 8-11mer peptides (*P* = 0.04) by unpaired t-test. There was no significant overall correlation of these increases in IgG and CD8+ IFNγ peptide recognition (**c**) for peptides unconfirmed by mass spectrometry (*P* = 0.75), but there was a significant correlation between improvements in IgG and CD8+ IFNγ peptide recognition (**d**) for mass spectrometry confirmed proteins (*P* = 0.01) by Pearson correlation coefficient. For 4T1 tumor recognition data previously presented in Fig. 5, WT and SNV antigens from proteins confirmed in 4T1 cells and vaccine by mass spectrometry (**e**) produced greater CD8+ IFNγ recognition of 4T1 cells if serum from those animals also had higher IgG recognition of those antigens

In order to confirm that we had observed stronger immune responses to proteins from our 4T1 vaccine, we performed quantitative tandem mass tag (TMT) liquid chromatography tandem mass spectrometry (LC-MS/MS) on both our 4T1 cell bank and the 4T1 autophagosome-enriched vaccine lots used in these studies to a depth of 4416 proteins. A number of the antigens studied in the T cell and IgG assays were confirmed as being found by TMT LC-MS/MS at a depth of 4416 confirmed tumor proteins. The results of this TMT LC-MS/MS data are available (Additional file 1: Data file S1). Although the observed increases in IgG recognition after vaccination did not depend on mass spectrometry identification for the 30 antigens investigated in T cell assays (Fig. 6a), or for other peptides on the IgG arrays (Additional file 2: Figure S5), there was an overall increase in CD8+ T cell recognition of WT and SNV 8-11mer peptides from proteins confirmed in the vaccine by TMT LC-MS/MS (Fig. 6b). These results translated to the IgG and CD8+ T cell correlation data presented previously (Fig. 4e), where we observed that the correlation between increased IgG signals and IFNγ release after vaccination occurred in the mass spectrometry positive fraction of the assays (Fig. 6c,d). Finally, we filtered the 4T1 tumor recognition data presented (Fig. 5a-d) for mass spectrometry identification, and observed a relationship between serum IgG signals against an antigen, and that antigen’s ability to improve recognition of 4T1 tumor. A more extensive breakdown of the individual results for mass spectrometry confirmed peptide recognition (Additional file 2: Figure S6) and tumor recognition (Additional file 2: Figure S7) is presented. These results suggest that the increased CD8+ T cell recognition observed is an antigen-specific response induced by our vaccine, which in turn correlates to an IgG antibody profile that strengthens, but also exists prior to treatment.

## Discussion

We observed IgG antibodies to 4T1 peptides in naïve female BALB/c mice, and this population of antibodies demonstrates stronger recognition of neoantigen peptides than autoantigen counterpart peptides which differ by a single amino acid. This result suggests a bias for the recognition of certain antigens prior to tumor exposure, perhaps caused by an individual’s unique history of tolerance to autoantigens or prior exposure to cross-reactive foreign antigens. Recent work has demonstrated a dramatic dependence of checkpoint blockade immunotherapies on specific microflora [38, 39], an effect typically ascribed to global cytokine changes, but perhaps also due in part to bacterial antigen cross-reactivity with tumor antigens. Germ free animals lacking such immunologic history do not respond well to cancer therapy [40]. The correlations between vaccine-induced T cell responses and antigen-specific IgG antibody signals observed in this study suggest that IgG may be one biomarker to observe such relationships between past immunologic history and future or ongoing anti-tumor immunity. And beyond the potential role of antibody as a biomarker, antibody may be directly involved in transferring prior immunologic knowledge to help prime or boost T cell populations.

Antigen-specific antibody can increase T cell activation through improved antigen uptake and cross presentation by antigen presenting cells in mice [41], and a similar Fc receptor dependent effect has also been observed in humans: some patients receiving monoclonal antibodies to EGFR (Cetuximab) generate elevated circulating EGFR_853–861_–specific CD8+ T cells [42]. Therefore, antibodies that happen to bind a tumor peptide or protein – such as those observed in this work – could provide a mechanism for improved CD8+ responses to those same antigens via increased cross-presentation efficiency. In addition to the overall trends we observed between IgG and CD8+ responses, we were struck that minimal peptides from Wdr33:H13Y – a mutation site with strong preexisting IgG signals in naïve animals – were recognized regularly in vaccinated animals by IgG and produced large increases in CD8+ T cell recognition of both 4T1 tumor and Wdr33:H13Y peptides. This exemplary case builds on our overall hypothesis that some antigen-specific CD8+ T cell responses to our vaccine are additionally favored by B cell and/or CD4+ recognition of similar peptides. While the mechanism is still uncertain, the observations reported here warrant further investigation into the roles of IgG and other antibody isotypes on future anti-tumor responses. Future work should focus on isotype-specific relationships and include data from clinical samples; this will be essential to determine these nuances of antibody and T cell interrelationships.

In the viral literature, the dependence of future responses on past ones to similar antigens is well documented [43–46], and there are recent reports suggesting that preexisting immunity not only occurs, but is common; many healthy adults have memory T cells reactive to peptides from viruses they have never encountered [47]. Perhaps such T cell repertoires relate to the observed universe of preexisting IgG autoantibodies common in humans [1]. If true, these IgG antibodies may help point to the types of antigens targeted by – or that evade – cancer immune surveillance. We may find that some patients require not just a release of tolerance or boosting of the natural priming environment by checkpoint blockade, but perhaps vaccines and therapies directed toward those antigens inadvertently avoided by their own unique history of antigen exposure. In the future, it may be possible to determine this antigenic history and correct gaps in immune surveillance with either personalized cancer vaccines or generalized complex vaccines like the autophagosome-enriched vaccine studied here.

## Conclusion

This study presents a simultaneous characterization of humoral and cellular immunity to tumor antigens at the level of individual peptides from a vaccine model whose components are currently undergoing human clinical trials [48, 49], and provides rationale for further investigation into the role of preexisting and post-treatment anti-tumor antibodies on anti-tumor CD8+ T cell immunity. Though this work focuses on neoantigens and autoantigens centered at mutation sites, these results may translate to other antigen populations, including non-mutated tumor antigens with ectopic expression patterns and antigens related to non-cancer diseases.

## Additional files

Additional file 1: **Table S1.** 4T1 Exome Overlap. Comparison of independent 4T1 whole exome sequencing analysis and prior literature. **Table S2.** Preliminary IgG Peptide Array. **Table S3.** IgG Peptide Array and ELISAs. **Table S4.** TMT Mass Spectrometry. (XLSX 2550 kb)

Additional file 2: **Figure S1.** Prophylactic autophagosome vaccination did not alter CD3 + CD4+ or CD3 + CD4 + FOXP3+ infiltrates from poly-I:C adjuvant only treatment. **Figure S2.** Overview of custom 4T1 mutation site peptide array. **Figure S3.** Vaccinated animals displayed increased IgG signals to 15mers and splenocyte IFNγ recognition of 15mer antigens. **Figure S4.** Increased IgG signal intensity to 4T1 15mers correlated with IgG signals in naïve animals. **Figure S5.** Antigens from proteins identified by mass spectrometry were not favored in IgG signal intensity increases. **Figure S6.** Increased post-vaccination IFNγ secretion in response to 4T1 mutation site peptides. **Figure S7.** Increased post-vaccination IFNγ secretion upon restimulation with 4T1 tumor cells. (DOCX 1638 kb)

## Abbreviations

LC-MS/MS: Liquid chromatography tandem mass spectrometry
SNV: Single nucleotide variant
TMT: Quantitative tandem mass tag
WT: Wild-type
