# Supplementary material for: Coordinated responses to individual tumor antigens by IgG antibody and CD8+ T cells following cancer vaccination
Source: J Immunother Cancer. 2018 Apr 5;6:27. doi: 10.1186/s40425-018-0331-0 (PMC5885379; doi:10.1186/s40425-018-0331-0)
Supplement: Supplementary file 2 — Figure S1. Prophylactic autophagosome vaccination did not alter CD3 + CD4+ or CD3 + CD4 + FOXP3+ infiltrates from poly-I:C adjuvant only treatment. Figure S2. Overview of custom 4T1 mutation site peptide array. Figure S3. Vaccinated animals displayed increased IgG signals to 15mers and splenocyte IFNγ recognition of 15mer antigens. Figure S4. Increased IgG signal intensity to 4T1 15mers correlated with IgG signals in naïve animals. Figure S5. Antigens from proteins identified by mass spectrometry were not favored in IgG signal intensity increases. Figure S6. Increased post-vaccination IFNγ secretion in response to 4T1 mutation site peptides. Figure S7. Increased post-vaccination IFNγ secretion upon restimulation with 4T1 tumor cells. (DOCX 1638 kb) [file 40425_2018_331_MOESM2_ESM.docx]

**Supplementary Materials:**


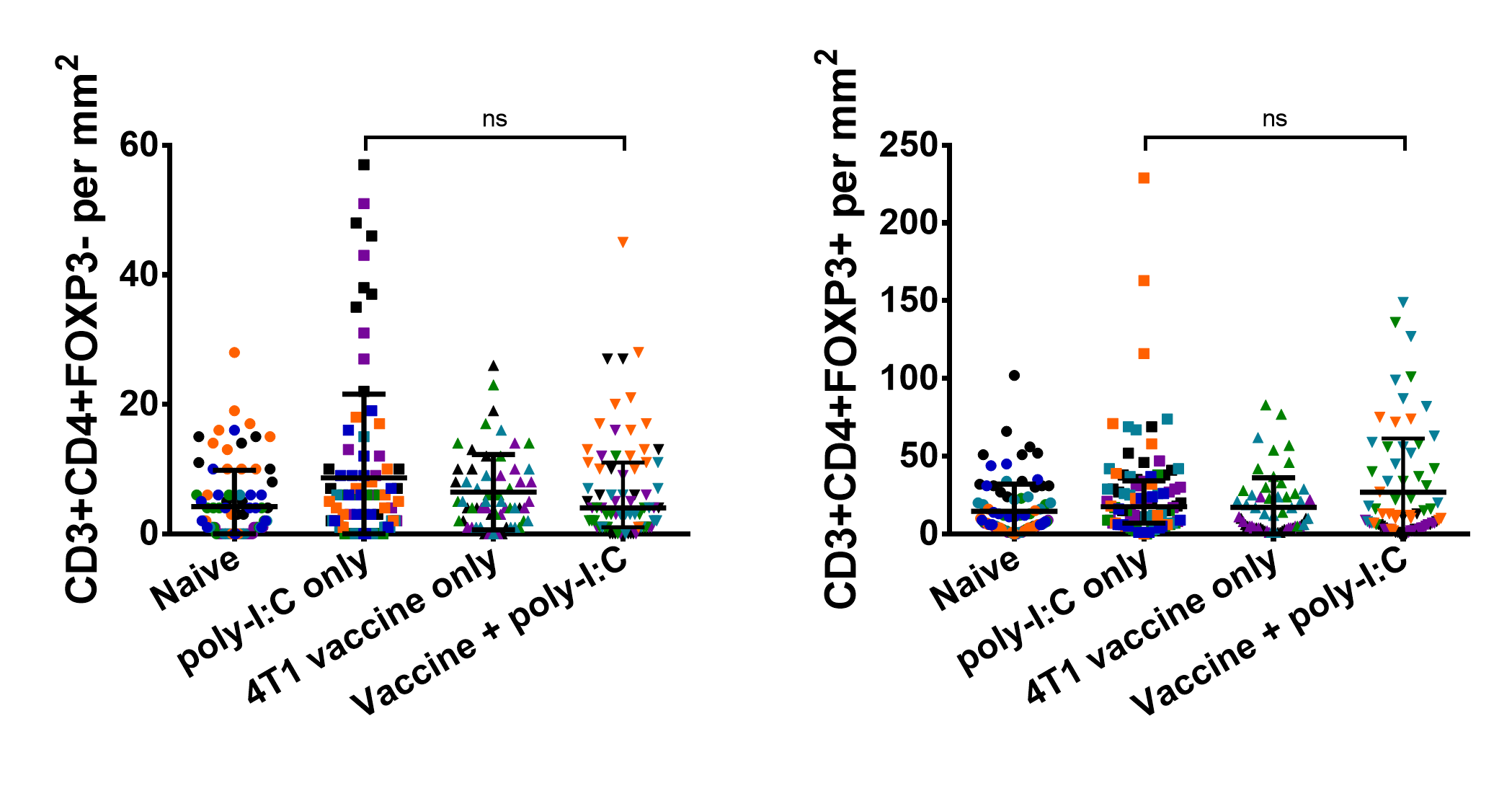


**B.**

**A.**

**Fig. S1. Prophylactic autophagosome vaccination did not alter CD3+CD4+ or CD3+CD4+FOXP3+ infiltrates from poly-I:C adjuvant only treatment.** Zinc and alcohol fixed day 30 4T1 tumors were stained for six color immunohistochemistry with tyramide signal amplification. (**A** and **B**) Fifteen fields were imaged for each of 4 to 6 tumors per group and quantified for (**A**) CD3+CD4+FOXP3- and (**B**) CD3+CD4+FOXP3+ per mm^2^ (fields from individual tumors colored separately). No significant difference in infiltrates were seen in the fields from 4T1 autophagosome vaccine + poly-I:C pretreated tumors versus poly-I:C only for CD3+CD4+FOXP3- infiltrates (P=0.29) or CD3+CD4+FOXP3+ (P=0.94) by t-test. Lines plotted are the median and interquartile range.

**
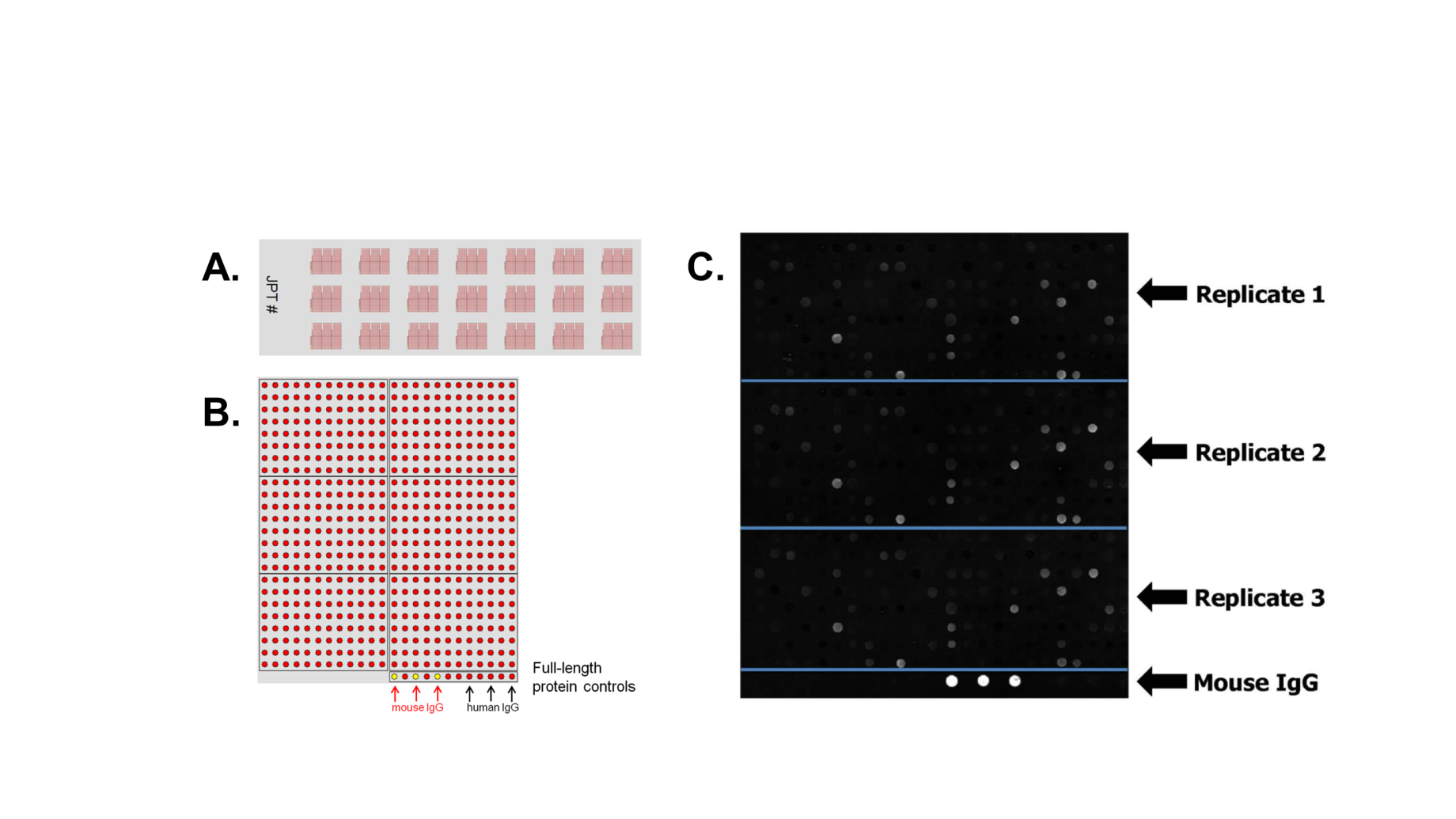
**

**Fig. S2. Overview of custom 4T1 mutation site peptide array.** Twenty arrays were printed by JPT peptides on a single slide with individual wells for each sample (**A**). These arrays consisted of AH1 plus 75 WT and 75 SNV 15mer peptides centered at 4T1 mutation sites. Peptides were printed in triplicate on each array along with anti-mouse IgG control spots (**B**). Whole mouse sera were pooled from three animals per experimental group, diluted 1:200, incubated on the arrays for one hour at 30 °C, and developed with an anti-mouse IgG secondary. All samples reacted to anti-mouse IgG control spots. Each spot was imaged with a high-resolution fluorescence scanner and quantified with spot-recognition software. Example image provided showing fluorescence from one of the 20 arrays printed for this experiment (**C**). Resulting values for each replicate were averaged across each of the three replicate spots, which then underwent interquartile range transformation prior to analysis.

**Data File S1**

**Tab 1: 4T1 Exome Overlap.** Comparison of independent 4T1 whole exome sequencing analysis and prior literature.

**Tab 2: Preliminary IgG Peptide Array.** Normalized data from custom 15mer peptide array of 4T1 SNVs and WT alleles centered at mutation sites identified by sequencing.

**Tab 3: IgG Peptide Array and ELISAs.** Raw data from custom 15mer peptide array of 4T1 SNVs and WT alleles centered at mutation sites identified by sequencing, paired with IFNγ release data to 31 different antigens in 8-11mer and 15mer lengths.

**Tab 4: TMT Mass Spectrometry.** Quantitative mass spectrometry used to profile 4T1 cells and vaccine.

**
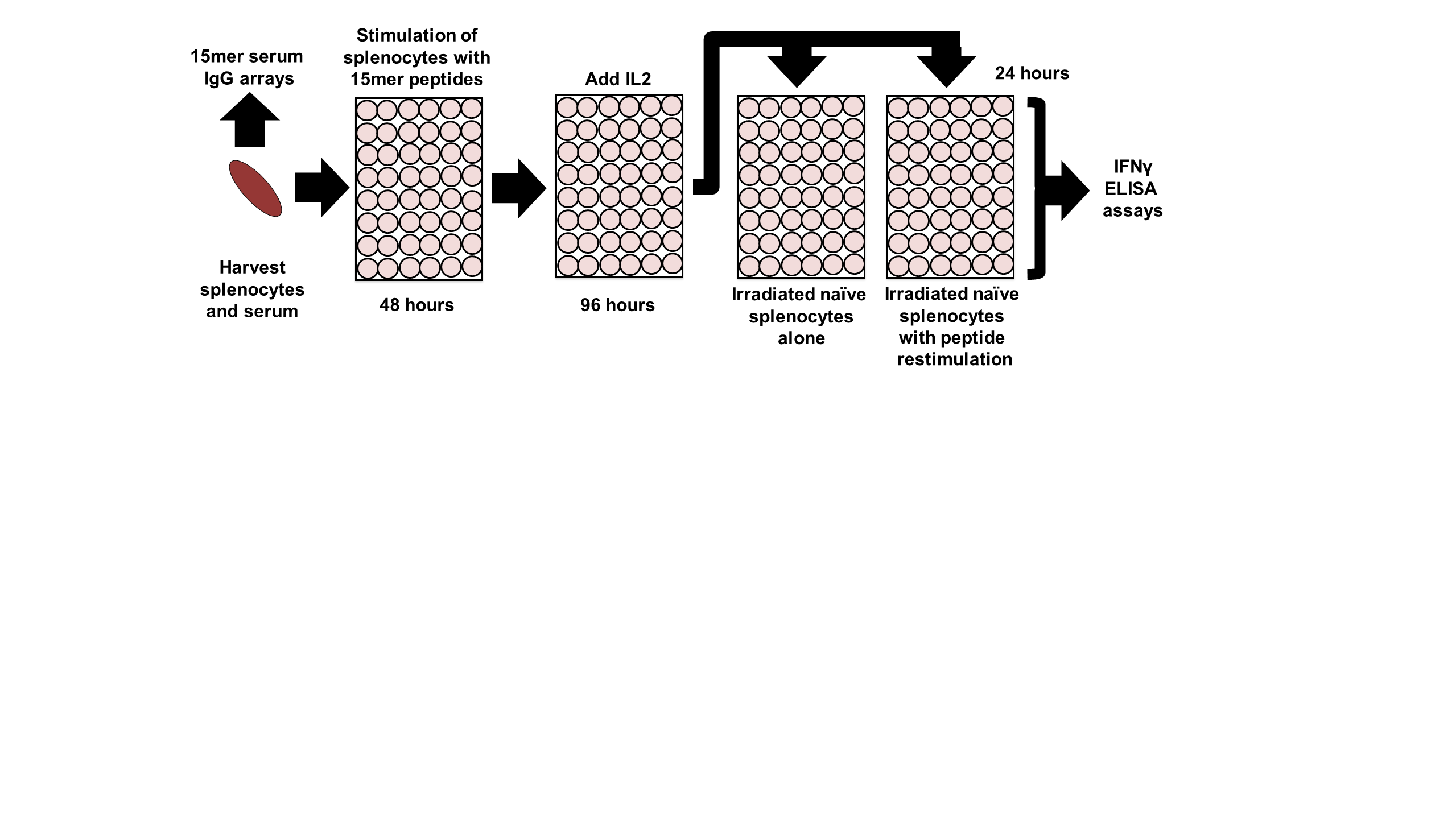
 Fig. S3. Vaccinated animals displayed increased IgG signals to 15mers and splenocyte IFNγ recognition of 15mer antigens.** Serum and splenocytes were harvested from naïve and 4T1 autophagosome vaccine + poly-I:C vaccinated animals. Serum was run on the 15mer arrays presented previously (Fig. 2). Splenocytes were stimulated with WT and SNV versions of 15mer mutation site peptides matching serum arrays for 48 hours, then expanded on IL2 for an additional 96 hours before wells were split and restimulated with either naïve splenocytes or naïve splenocytes pulsed with a second stimulation of peptide. Graphs are of the average increase in IFNγ secretion by ELISA in wells with peptide restimulation over splenocytes alone for n=3 experiments with vaccine groups and n=2 experiments with naïve groups. (**A,B**) Increase in average IFNγ secretion in vaccine groups upon secondary exposure to n=15 different WT (P=0.005) (**A**) but not n=15 different SNV peptides (P=0.25) (**B**) by Wilcoxon matched-pairs signed rank test. (**C,D**) Simultaneous serum IgG array recognition data for the same 15mer peptides from the same n=3 vaccine groups and n=2 naïve groups used in splenocyte assays. Increase in average IgG signal intensity to n=15 different WT (P=0.01) (**C**) and n=15 different SNV (P=0.02) (**D**) peptides by Wilcoxon matched-pairs signed rank test. (**E**) Combined data previously presented in (**A-D**) plots average differences in IgG and IFNγ recognition for each of the n=15 WT and n=15 SNV mutation sites. Positive values represent increased signals in vaccine groups and negative values represent increased signals in naïve groups. Values in upper-right quadrant demonstrated simultaneous increases in IgG and splenocyte IFNγ recognition of individual 4T1 mutation-site antigens in vaccine groups. However, there was no significant overall correlation of these increases in recognition (P=0.5) by Pearson correlation coefficient.

**D.**

**C.**


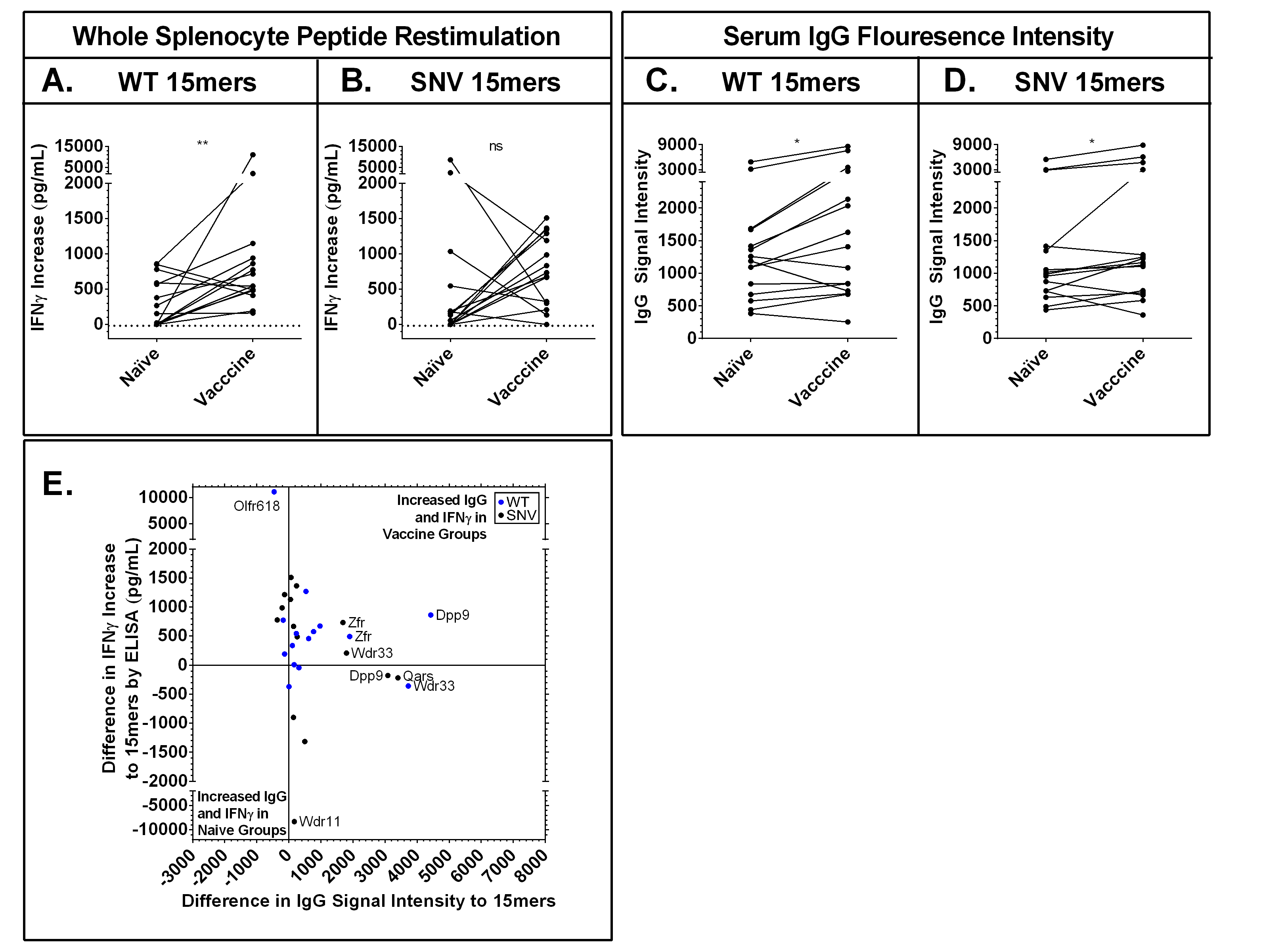


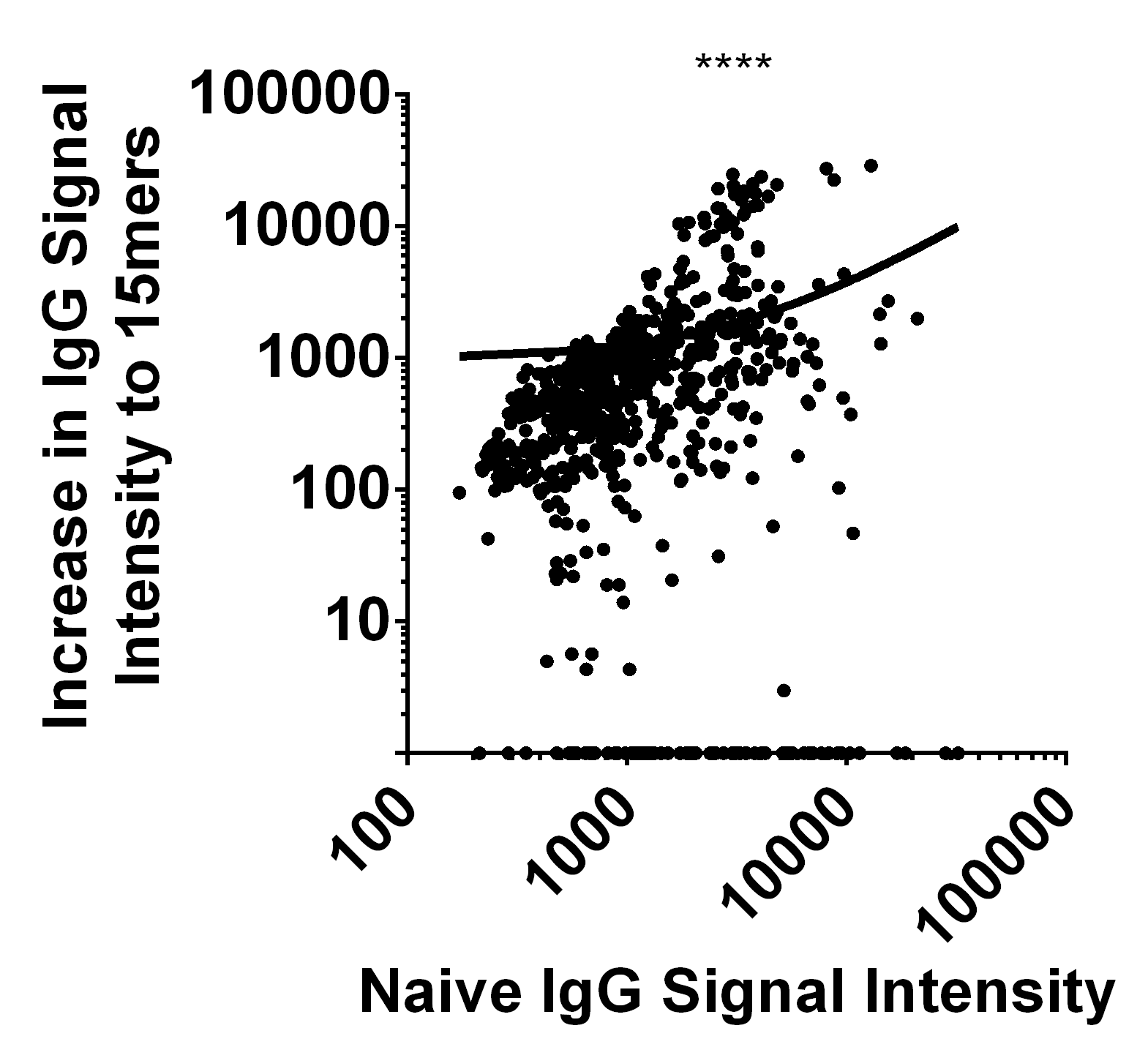


**Fig. S4. Increased IgG signal intensity to 4T1 15mers correlated with IgG signals in naïve animals.** Data are from five independent pairs of IgG arrays presented previously. These arrays were constructed with 15mer peptides matching 75 WT autoantigens paired with 75 SNV neoantigen mutation-sites in 4T1 plus AH1, and were reacted with pooled serum from naïve and autophagosome-enriched vaccine + poly-I:C treated mice. Data compare the positive increase in IgG signal intensity after vaccination to the IgG signal intensity in naïve animals for each of n=755 paired points, and are plotted in log­_10_ scale. There was a significant overall correlation (P<0.0001) by Pearson correlation coefficient.


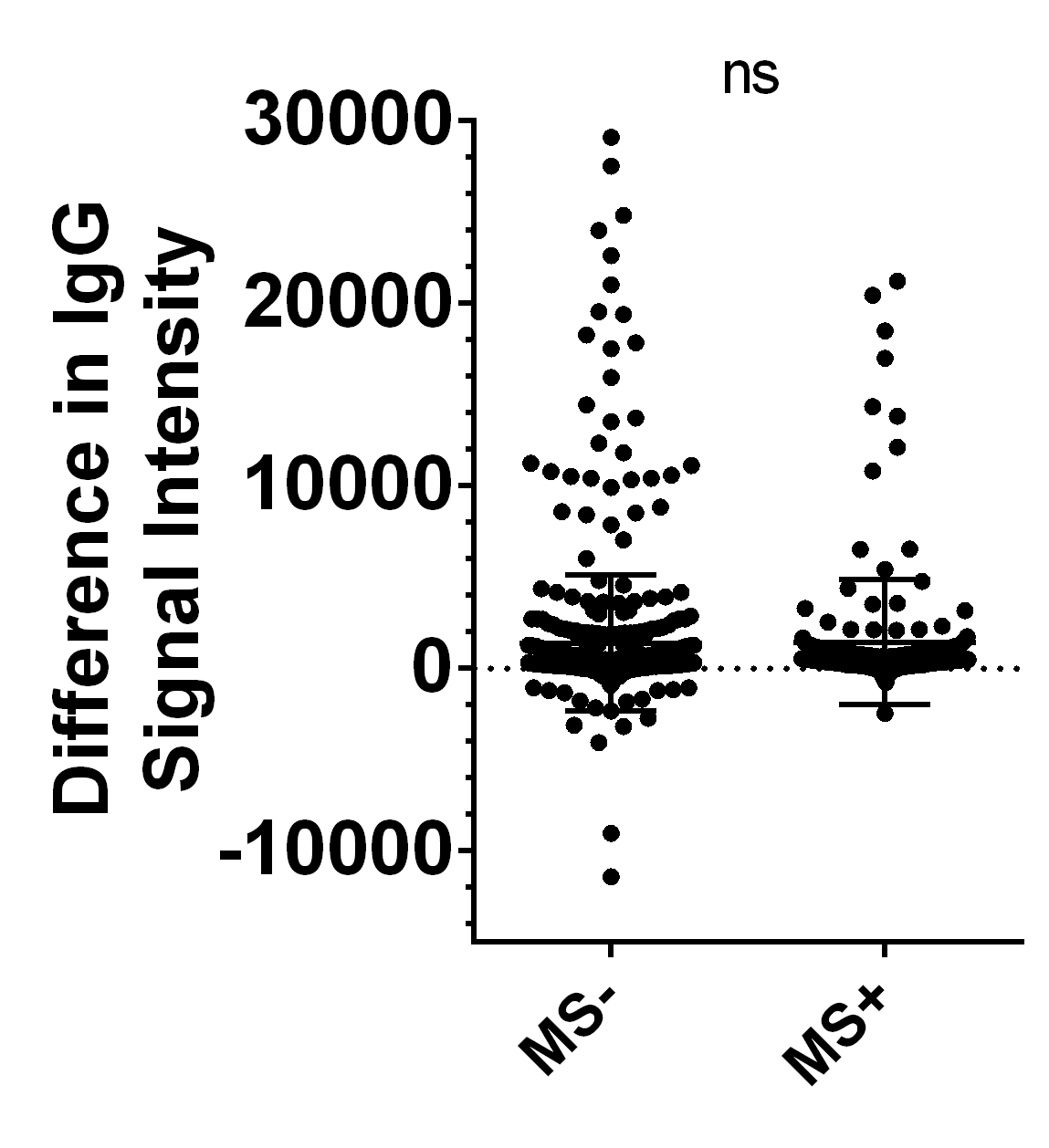


**Fig. S5. Antigens from proteins identified by mass spectrometry were not favored in IgG signal intensity increases.** Data are from five independent pairs of IgG arrays presented previously. These arrays were constructed with 15mer peptides matching 75 WT autoantigens paired with 75 SNV neoantigen mutation-sites in 4T1, and reacted with pooled serum from naïve and autophagosome-enriched vaccine + poly-I:C treated mice. Data compare the difference in signal intensity for 15mer proteins either confirmed, or not confirmed, in the vaccine by mass spectrometry. There was no significant increase in signal intensity to 15mer peptides from proteins confirmed in 4T1 vaccine by mass spectrometry (P=0.61) by Mann Whitney test.


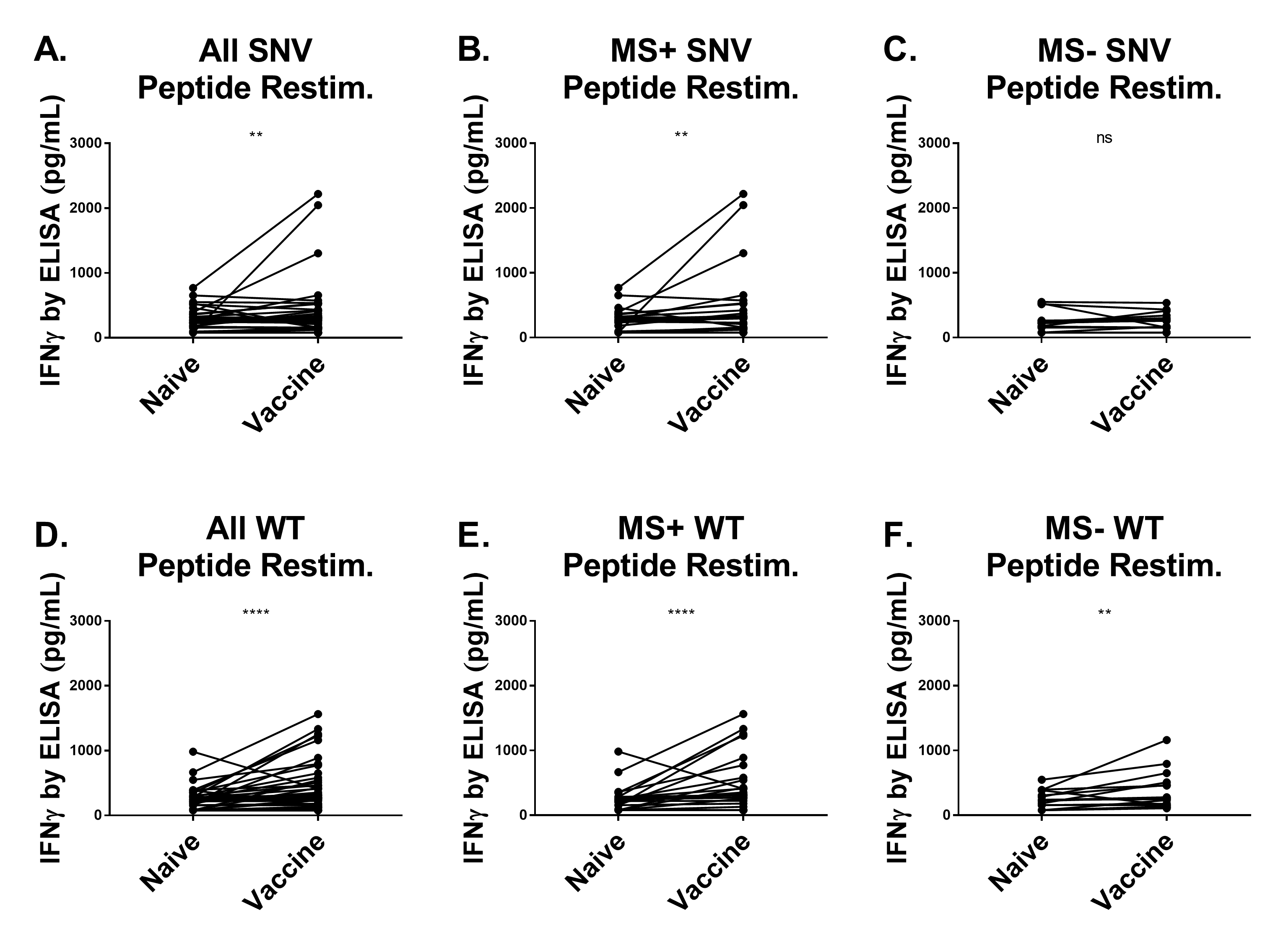


**Fig. S6. Increased post-vaccination IFNγ secretion in response to 4T1 mutation site peptides.** Individualized data from all three sets of paired experiments with breakdown by mass spectrometry identification status presented (Fig. 4). CD4 depleted splenocytes from naïve and 4T1 autophagosome-enriched vaccine + poly-I:C vaccinated animals were stimulated with WT and SNV versions of top predicted MHCI binding 8-11mer mutation site peptides, then expanded on IL2, before being washed, split, and restimulated with naïve irradiated splenocytes and peptides. (**A**) Increased IFNγ secretion in vaccine groups upon secondary exposure to n=15 SNV peptides regardless of LC-MS/MS confirmation (P=0.0015). (**B**) Increased IFNγ secretion in vaccine groups upon secondary exposure to n=9 different SNV peptides from LC-MS/MS confirmed proteins (P=0.0031). (**C**) No difference in IFNγ secretion was observed in vaccine groups upon secondary exposure to n=6 different SNV peptides from LC-MS/MS unconfirmed proteins (P=0.22). (**D**) Increase in IFNγ secretion in vaccine groups observed upon secondary exposure to n=15 WT peptides regardless of LC-MS/MS confirmation (P<0.0001). (**E**) Increase in IFNγ secretion in vaccine groups observed upon secondary exposure to n=9 different WT peptides from LC-MS/MS confirmed proteins (P<0.0001). (**F**) Increased IFNγ secretion in vaccine groups observed upon secondary exposure to n=6 different WT peptides from LC-MS/MS unconfirmed proteins (P=0.0010). All statistics by Wilcoxon matched-pairs signed rank test.

**C.**

**D.**

**A.**

**B.**


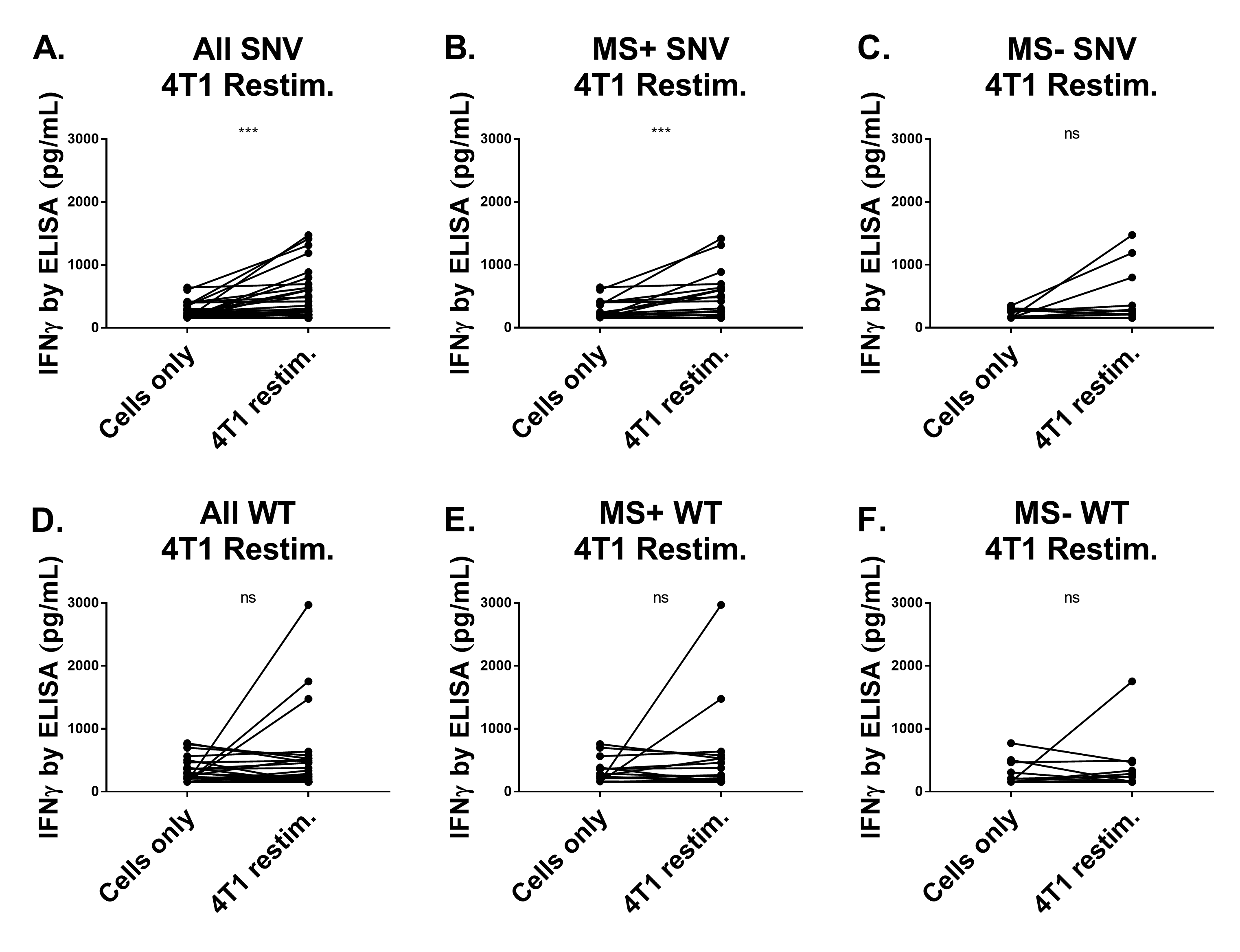


**Fig. S7. Increased post-vaccination IFNγ secretion upon restimulation with 4T1 tumor cells.** Individualized data from both sets of paired experiments presented with breakdown by mass spectrometry identification status (Fig. 5). CD4 depleted splenocytes from naïve and 4T1 autophagosome-enriched vaccine + poly-I:C vaccinated animals were stimulated with WT and SNV versions of top predicted MHCI binding 8-11mer mutation site peptides, then expanded on IL2, before being washed, split, and restimulated with live 4T1 cells or placed into empty wells with media alone. (**A**) Increase in IFNγ secretion in vaccine groups observed upon secondary exposure to 4T1 after primary exposure with n=15 SNV peptides regardless of LC-MS/MS confirmation (P=0.0002). (**B**) Increase in IFNγ secretion in vaccine groups observed upon secondary exposure to 4T1 after primary exposure with n=9 different SNV peptides from LC-MS/MS confirmed proteins (P=0.0004). (**C**) No difference was observed in IFNγ secretion in vaccine groups upon secondary exposure to 4T1 after primary exposure with n=6 different SNV peptides from LC-MS/MS unconfirmed proteins (P=0.148). (**D**) No difference in IFNγ secretion in vaccine groups was observed upon secondary exposure to 4T1 after primary exposure with n=15 WT peptides regardless of LC-MS/MS confirmation (P=0.65). (**E**) No difference in IFNγ secretion in vaccine groups upon secondary exposure to 4T1 was observed after primary exposure with n=9 different WT peptides from LC-MS/MS confirmed proteins (P=0.597). (**F**) Increase in IFNγ secretion was seen in vaccine groups upon secondary exposure to 4T1 after primary exposure with n=6 different WT peptides from LC-MS/MS unconfirmed proteins (P=0.99). All statistics by Wilcoxon matched-pairs signed rank test.

**C.**

**D.**

**A.**

**B.**
